# Experiences of people with memory disorders and their spouse carers on influencing formal care: “They ask my wife questions that they should ask me”

**DOI:** 10.1177/1471301221994300

**Published:** 2021-02-17

**Authors:** Mari S Aaltonen, Anne Martin-Matthews, Jutta M Pulkki, Päivi Eskola, Outi H Jolanki

**Affiliations:** Faculty of Social Sciences, (Health Sciences), and Gerontology Research Center (GEREC), 7840 Tampere University, Finland; Department of Sociology, University of British Columbia, Canada; Department of Sociology and Office of the Vice-President, Health, University of British Columbia, Canada; Faculty of Social Sciences, (Health Sciences), and Gerontology Research Center (GEREC), 7840 Tampere University, Finland; Faculty of Sport and Health Sciences, Gerontology Research Center (GEREC) and Open University, University of Jyväskylä, Finland; Faculty of Social Sciences, Health Sciences, and Gerontology Research Center (GEREC), 7840 Tampere University, Finland; Department of Social Sciences and Philosophy, 4168 University of Jyväskylä, Finland

**Keywords:** memory disorder, agency, family care, informal care, formal care

## Abstract

**Background:**

People with memory disorders often need care and help from family carers and health and social care providers. Due to the deterioration of cognitive capacity and language skills, they may be unable to convey their thoughts and care preferences to other people. As a result, their agency may become restricted. We investigated the descriptions provided by people with memory disorders and spousal carers of their influence on care in encounters with formal care providers.

**Methods:**

Qualitative thematic analysis was used to identify, analyze, and report themes that describe encounters with professionals in different social or healthcare environments. In-depth interview data were gathered from 19 spouse carers and 15 persons with memory disorders.

**Findings:**

Three themes out of four describe how people with memory disorders and their spouse carers influence formal care: Acquiescence, negotiating care decisions, and taking control. The fourth theme describes lack of influence. People with memory disorders and their spouse carers have ways to influence care, but spouse carers identified more ways of doing so. Both either accepted and followed the care guidelines by the formal carers or took control of the situation and made their own decisions. Spouse carers also sought to influence care decisions through negotiations with formal carers. When formal carers’ decisions were experienced as inconsistent or the rationale of their actions difficult to follow, the possibilities to influence care were limited.

**Conclusions:**

People with memory disorders and their family carers are often in a disadvantaged position as they lack power over the health and social care decision-making during the illness, which is often guided by structural factors. To support the agency of people with memory disorders and to promote shared decision-making, clarification of the service structure and clearer communication between the different parties involved in care are required.

## Introduction

*We, people with memory problems, may experience that we are not always treated seriously or sensitively, but ignored. This can be done even though there is nothing wrong with our sanity or minds, but our short-term memory and maybe our sense of direction are damaged. Surely each of us has sometimes experienced some degree of rejection, and we know how it hurts. When I say that I have a memory disorder, I have noticed that some people then ask my wife questions that they should ask me. We need help, but you can do it without having to make a song and dance about it* (Erkki, person with memory disorder, in a written text provided to the interviewer).

Given the complex and increasing care needs that people with memory disorders often have, care and help are usually needed from both family carers and various health and social care providers (Jensen & Inker, 2015; Robinson et al., 2010; Teel & Carson, 2003). In addition, family carers often need formal-sector support to deliver care at home (McCabe et al., 2018; Sims-Gould & Martin-Matthews, 2010). This diversity and multiplicity in actors involved in care and everyday life can increase the difficulties of people with cognitive impairments to influence their care and thus restrict their agency. In this study, we investigate how people with memory disorders and their spouse carers describe their possibility to influence the care provided by formal care providers to the person with memory disorder. With care, we refer broadly to any type of formal care and assistance received from the social and healthcare professionals. We chose to use the terms *memory disorder* and *people with memory disorders* to refer to people who have been diagnosed with a progressive disease that impairs memory and other cognitive capacity, but not everyone has their condition progressed to dementia yet. Thus, memory disorder is the best term to be used in this study to cover the diversity of the interviewees’ cognitive health status.

In this study, we have adopted agency as an orienting concept for a number of reasons. First, having an influence on one’s personal circumstances is considered a part of human agency (Boyle, 2014) as is the ability to make decisions (Bosco et al., 2019). Sense of agency refers to the ability to control personal life in a meaningful way (Kitwood & Bredin, 1992). Due to deterioration of cognitive capacity and language skills, people with dementia may struggle or be unable to convey their thoughts and care preferences to other people (Boyle, 2014). As a result, their agency—and the possibilities to influence their care—may become restricted (Groen-van de Ven et al., 2018). Second, agency is a crucial part of well-being at every stage of life, even in advanced dementia, and the struggles to maintain agency might be interpreted as challenging behavior in dementia (Kitwood & Bredin, 1992). However, there is evidence that recognizing the agency of people with memory disorders has positive outcomes: It increases their social action, helps to restrain the adverse effects of cognitive decline (Bosco et al., 2019), enhances their autonomy, and improves their quality of life (Feinberg & Whitlatch, 2001). Third, according to previous research, people with dementia wished to remain central in decision-making as long as possible (Fetherstonhaugh et al., 2013), and when their role in decision-making was reduced or removed, they felt marginalized and excluded. Supportive decision-making (e.g., Sinclair et al., 2019) and advanced care planning (e.g., Dickinson et al., 2013) can be made during the early stage of the condition. Nevertheless, various decisions concerning daily care are made in encounters and interactions with formal carers. These decisions cannot be anticipated in advance but arise in the course of the illness.

Informal care has always played an essential role in care for older people, even in Finland, with a strong public care sector (Kehusmaa, 2014). In Finland, the family has no legal obligation to provide care as this responsibility rests on local authorities, that is, municipalities (Keskimäki et al., 2019). Formal carers for people with memory disorders usually include a variety of professionals from health and social care services. The provision of social care services, for example, home care and institutional long-term care and respite care (the latter intended for a family carer’s rest and varies from few days to few weeks), is the responsibility of municipalities. Municipalities and hospital districts provide inpatient and outpatient health care and medical services (Keskimäki et al., 2019).

Family members are important not only as providers of care and support but also as contributing to the agency of people with memory disorders (Bosco et al., 2019). For frail older adults who have reduced ability to influence their personal situation, a family member can help by making their putative views and wishes visible (Lambotte et al., 2019). Thus, the informal carer can act as a “substitute agent” or use their agency for the benefit of the person who receives the care. These require that the informal carer has knowledge and recognition of his/her wishes and preferences. Informal care for a person with dementia requires collaboration with formal carers to gain support adjusted to the specific needs at different stages of dementia (Lethin et al., 2016). A situation where informal and formal carers share the responsibility for care (Sims-Gould & Martin-Matthews, 2010) can lighten the work of family carers. Family carers who perceive a higher level of collaboration with nurses show a higher degree of preparedness for caring at home (Hagedoorn et al., 2020). However, the collaborative relationship does not always work ideally, and poorly executed encounters with formal carers can add to family carers’ stress (Laparidou et al., 2019; Peel & Harding, 2014; Thoma-Lürken et al., 2018).

In this study, we explore *the descriptions provided by people with memory disorders and spousal carers of their influence on care in encounters with formal care providers*. The study aims to detect different ways people with memory disorders and spousal carers strive and are able to influence formal care. In addition, we aim to recognize *situations where their influence on care is described as restricted or even nonexistent*. We begin from the idea that the ability to make decisions and to express one’s own will to other people is an integral part of agency, but influencing care is also likely to include other types of effort than just decision-making. Rather enactment of agency requires actions from the parties involved. Thus, “influencing care” ideally mean that people receiving care and their spouses have an opportunity to express their will and change the content, quality, and delivery of care, if it does not meet their needs and expectations. Hence, it is essential to remain sensitive in the analysis to the interviewees’ different ways to express their wishes and hopes regarding care.

## Data and methods

Data come from interviews of people with memory disorders and their spousal carers. From here on, when we talk about people with memory disorders and their spouses together, we refer to them as *dyads*. We recognized the possible difficulties of people with memory disorders in responding to interview questions, and for this reason, we conducted the interviews mainly as dyad interviews. In this way, both parties had an opportunity to express their views, but, if necessary, the spouse was able to support the person with the memory disorder during the interview. The analysis focuses on their descriptions of different encounters with formal care providers. We use “formal carer” in its broadest sense as descriptions do not necessarily provide information on a specific provider sector (health or social care), and the descriptions of care encounters with different formal carers may overlap or diverge.

Altogether, 19 interviews were collected from 34 interviewees in Finland between October 2018 and March 2019. The dyads were free to choose whether to be interviewed together or separately, in a place convenient to them. In most cases, participants (26 persons/13 dyads) chose to give their interviews together in their own homes. Two dyads (4 persons) felt that they could express themselves more freely if they were interviewed separately. In four cases, only the carer was interviewed. In three of these cases, the spouse with a memory disorder was not able to communicate his/her experiences. One person with memory disorder did not want to participate himself but did not oppose the spouse’s participation. The health conditions of the interviewees with memory disorders varied from mild to severe according to their own perception, but everyone was in sufficiently good health to give informed consent and participate in the discussion.

Interviews were conducted once with each dyad (or with a spousal carer in those four cases in which the person with memory disorder was not able to participate), audio-recorded and transcribed verbatim. The participants were recruited with help from the Alzheimer Society of Finland and the Carers Finland. The interested participants contacted the researchers themselves.

In the thematic, in-depth, semi-structured life-course interviews, the interviewees were asked to describe which health care and social care services they had used and whether they had been content with the help and care received. In addition, they were asked if there was some type of help or care they felt they needed but did not receive. By describing their views on these issues, the interviewees discussed the content and quality of and satisfaction with care in encounters with formal carers in different situations. While discussing different aspects of care and formal care services, the interviewees also expressed their views on the possibilities to influence formal care and different situations in which this had succeeded or failed. In all, the interviewees were encouraged to direct the discussion and raise any issues they considered particularly important.

### Ethics

The research was undertaken in several regions located in Finland. Ethical approval was obtained from The National Ethics Committee of the Tampere Region (Decision 37/2018). Each participant gave their independent informed consent to the interview and its recording. The participants were informed of their option to cancel the interview at any point and to refuse permission to use the research data thereafter. The participants were also informed of the data collection and handling procedures. The interviews were conducted by two of the authors. All research procedures were conducted according to the General Data Protection Regulation (European Commission, 2018). To ensure the privacy and confidentiality of the interviewees, pseudonyms are used.

### Qualitative analysis

We followed the guidelines and phases for thematic analysis outlined by Braun and Clarke (2006). Thematic analysis is a method for identifying, analyzing, and reporting patterns (themes) within data (Braun & Clarke, 2006). We analyzed all talk that mentions any encounters with professionals in different social or healthcare environments. We first assembled initial codes (Braun & Clarke, 2006) from the data. In our analysis, the codes illustrate accounts of approaches that the interviewees had adopted to influence care in encounters with the professionals. The codes included acceptance, trust, consent, negotiation, persuasion, convincing, seeking care, persistency, conflict, rejection, powerlessness, and confusion. Data were coded separately for people with memory disorders and their spouse carers to see how their accounts varied. Next, codes were organized into broader themes. According to Braun and Clarke (2006), a theme represents a patterned response. Our themes are based on their similar purpose in encounters with formal carers. The first three themes—*acquiescence*, *negotiating care*, *and taking control*—are linked to various situations in which people with memory disorders and their family carers described themselves to be able to influence care provided to the person with the memory disorder. Interviews also include accounts that describe situations where they found it difficult to influence formal care. These accounts were classified under the fourth theme *lack of influence*. The themes partly overlap and often build on each other. A description of the study population and the themes is presented in Table 1.

**Table 1.** Description of the study population and themes.

	Persons with memory disorders	Spouse carers
*N* (%)	15 (44)	19 (56)
Sex (%)		
Men	9 (26)	6 (18)
Women	6 (18)	13 (38)
Age (mean)	76	75
Interviewed (%)		
Alone	2 (6)	6 (18)
Dyad	13 (38)	13 (38)
Interviewed (%)		
At home	14 (41)	16 (47)
At the Alzheimer Society	1 (3)	3 (9)
Broader themes (codes), interview includes one or more account with the theme (% of all interviewees)		
1. Acquiescence (acceptance, trust, and consent)	8 (24)	14 (41)
2. Negotiating care (negotiation, persuasion, and convincing)	0 (0)	10 (29)
3. Taking control (seeking care, persistency, conflict, and rejection)	6 (18)	16 (47)
4. Lack of influence (powerlessness and confusion)	6 (18)	12 (35)

## Findings

### Acquiescence

Acquiescence includes excerpts where interviewees expressed their *acceptance* of the hierarchy between the dyad and professionals and *trust* in the decisions by professionals. Accepting the decisions requires *consent* to the proposed actions. In some decisions, the person receiving care can have very little say such as giving up one’s driving license due to deteriorated cognition. However, in accounts expressing acquiescence, people are not forced to follow professionals’ decisions, but they choose to do so.

It was typical that the interviewees did not clearly express how they felt about the course of action—whether they felt the procedures are adequate, necessary, or redundant. In some cases, the interviewees followed the decisions made by professionals even though they did not like them. In the extract below, Ilpo, a person with a memory disorder, describes how unpleasant memory tests are. However, he did not refuse to take the test or oppose the nurse.

*Irma (spouse): And you do the memory test there [during the appointment with the nurse] every time.*

*Ilpo: The same [test]. In principle I do get the help I need, I’m not saying that. But that… There will be no cure or recovery from this, that is a problem to me.*

*I feel bad that I have to answer those questions [in the memory test], do those things they ask me to do, you know, I don’t, I won’t recover from this illness, ever.*

In the following extract, Valma, a spouse carer, describes how she was encouraged to learn how to catheterize her husband. She talked a lot about the hygiene and bladder problems and troubles they had coping at home, but she did not mention how she felt about catheterizing her husband. Yet she accepts the nurses’ decision to teach her and does not oppose them.

*Q: Who taught you catheterization?*

*Valma: At the primary care hospital they said “yes you will learn it”. And one day I went there, they showed me how to do it. It’s after all… they have those disposable catheters, it’s after all a simple thing to do.*

### Negotiating care

Some accounts refer to situations where negotiations guide care. None of the people with memory disorders themselves provided these accounts, only the spouse carers. Negotiations can be divided into two types: those in which the desire to *negotiate* is mutual, that is, shared decision-making and to those in which the spouse carer tries to *persuade* or *convince* the formal carer of his/her own view on what is the proper care. In the first type of negotiation, care is planned in collaboration and mutual understanding with the formal carer, and the spouse carer’s influence over care is substantial. Tuomas (spouse) describes how the doctor does her best to hear and take into account his wife’s views on things and simultaneously respects the spouse’s perceptions.

*Tuomas: This female doctor is very nice. She also talks about everything else, thus it is very easy to note that she [the wife with the memory disorder] can, she is able to, to [answer] some questions of hers.*

…………………………….

*Tuomas: We agreed with [the doctor]. Can you imagine? She respected my opinion and then, well, started to reduce [the number of medications].*

Sometimes, the negotiation starts from a disagreement. It may be that spouse carers have to negotiate with or *persuade* many formal carers until they find someone—an ally—who will listen to their side of the situation and steers the care toward spouse’s preferences. Next, Tyyne (a spouse) describes negotiating care with an ally.

*Tyyne: The diabetes nurse was the one who started the process, we visited there a little while ago, and of course I said that I was desperately hoping [for a neurologist appointment] but no, no, no, there is no possibility to reach the physician [for a referral to a neurologist]. She says she will put it in his file. And the doctor called. So this was definitely organized by her [the nurse]. Without her we didn’t have any chance [to get a doctor’s referral to a neurologist].*

The data showed the complexity of care situations and that the views and preferences of the dyad do not always meet. Sometimes, spouse carers relied on private communications with formal carers to *convince* them of their views, which in fact differ from the perception of the person with the disorder. Spouse carers talked about how formal carers are not able to see the true nature of all the symptoms, and as a result, they felt the need to express their views in the absence of their spouse. Maire (a spouse) was deeply concerned because her life partner still had a valid driving license, but she felt he was in no condition to drive anymore. However, Markku, a person with memory disorder, disagreed and became upset if she talked to the doctor about it. She, therefore, contacted the doctor before Markku’s appointments.

*Maire: I said before the doctor came that, that, it is not possible to talk about this in the presence of Markku, talk about how things really are. Because he gets mad*[…]* I should talk to the doctor before this appointment starts, and immediately they said that the person concerned must be present. Well, I’m stubborn, and I walked down the hall and was able to talk to them then. I mean, this situation is like, the case is that other people’s lives are already in danger.*

…………………………………………………………..

*Maire: I went out for a walk, the doctor then called me so that I could talk freely*[…] *I told that doctor too that I feel awful about talking that way, behind his back. But I have to.*

### Taking control

A clear and direct way to influence care was to take control over the care by seeking care, persistently demanding care, conflicting with formal carers, and rejecting the help. First, interviewees presented different ways of *seeking care*: at the early stage of condition to find adequate services and later other types of care if the previous option does not seem adequate or sufficient. In the next extract, Anja (a person with a memory disorder) and Aulis (a spouse) explain how they—especially Anja—have sought proper care from several possible care providers. Their conclusion is that even if one is diagnosed and “in the system,” one needs to be active and take care of oneself.

*Aulis: They had already received all the information on her. Neither the memory coordinator nor the nurse specialized in memory issues contacted us. I was just thinking let’s see how long it takes. But then she called herself and went…*

*Anja: I couldn’t wait any longer.*

*Aulis: She went there, was it last week or the week before that… I mean, these social and healthcare services, even though you have the diagnosis. Of course, we are probably more active than others, but what about if you are an ordinary Joe Schmo, and you don’t know this system or nothing about these services, then you are left entirely [alone].*

Even though formal carers hold power over various care procedures, the interviewees do not necessarily accept this. They disagreed with decisions, and they doubted whether they were receiving the care they need. In these cases, the interviewees lack trust in formal carers, and they feel that formal carers’ actions do not reflect their expectations and needs. In the next extract, a spouse carer Lasse *persistently* seeks and demands care.

*Lasse: There’s always a different doctor that sees you [in emergency room]. The best weekend was when we went there, when the fever was high and my wife was in pain, and she had been discharged from the hospital, and it only took one day. On Friday I took her quickly to the ER [emergency room], and they monitored her there for a while and then said to come and pick her up, and if you’re not coming, we’ll put her in a taxi, and I said no, you don’t put her there*[…]* The next day I went there again, this time [to the ER] in an ambulance. It was one night there again, and then she was sent back home. The third time I took her there again on Sunday night, Monday morning, then I said I’m not going to take her back if you don’t now... take her somewhere. Then she was admitted.*

……………………………………………………………………………….

*But in the public sector, you don’t get [care] if you don’t ask. So you have to always ask, so that’s it…. if you just have the courage and, well, you ask so many times then … you probably get information if you just know how to ask.*

Sometimes, the disagreement escalates into *conflict* when the person with the memory disorder or the spouse carer decides to *reject* help from a particular formal care provider. In these accounts, the interviewees brought forward their view that the care was so dysfunctional or redundant that they were better off without it. In the next extract, Tuomas (a spouse) describes the care facility where his wife Tiina was in respite care and his dissatisfaction with care. Later, he explains that he decided never to take her there again. In the following extract, Ville, a person with memory disorder, describes how he decided not to stay in the nursing home during weekends as he considered it redundant.

*Tuomas: If they take her there again… it will be the same again, they just medicate her so that she doesn’t know anything, and just put the diapers on. They don’t do anything extra there. She didn’t recognize me. I said to them, now you have medicated her too much. And I was right. When I called them that night, they had all that on their computer.*

*Q.: After that you haven’t had these respite care episodes?*

*Tuomas: No, we haven’t, and I will never take her there again. It was a mistake in the first place. But in a way it was good, I got to see what it was like.*

…………………………………………………

*Valma (spouse): They didn’t want to let him come home.*

*Ville: There wasn’t anything happening during the weekend, no treatments, then I said, “Well, can’t I just go home?” And they said, “What type of bathroom do you have there, can you manage there?” Then I said, “Well, hell, we have the kind of bathroom you should have here.” I then traveled so that I was at home every weekend, as a trouble to my wife and children.*

### Lack of influence

It was relatively common for interviewees to describe situations in which they have very little or no influence over formal care. Interviewees express *powerlessness* when their perceptions of the necessary care conflict with the perceptions of professionals. The dyads feel *confused* about the care. They do not have the power to direct the care as they wish because the formal carer has the power to disregard their perceptions of what should be done. In the next extract, Tyyne explains how they first had contact with a neurologist, but then it ended at the doctor’s initiative. In the subsequent extract, spouse carer Taina describes powerlessness when formal carers disregarded her husband’s needs in the hospital. Both extracts imply that the rationale of the formal carer’s actions was not explained for the spouse or the person with memory disorder.

*Tyyne: With this disorder we haven’t received anything after the, the visits to the neurologist ended. It was quite, it was quite nice. It was like a safe thing. But no, after that, no.*

…………………………………………….

*Taina: Then he received the, the report about what had been done. It read that he must drink and eat by himself, that this... it wasn’t a drip, but I don’t remember what they call it now, hydration. There’s no follow-up treatment. Meaning that was a death sentence [talks quietly, become sensitive]. Because it wasn’t possible for him to eat or drink. He weighed 47 kilos at that time.*

People with progressive memory disorders often are at a very vulnerable position: It is their lives that are affected by care decisions, but at the same time as their condition progresses, their symptoms add to the confusion. Sometimes, the descriptions of a lack of influence on care reveal very complicated situations: A combination of lack of mutual understanding, unclear communication, and power imbalance between the dyad and the formal carer(s). In the following extract, the spouse carer Kaisa describes a situation in which all these elements are intertwined.

*Kaisa: I was so sorry when this medical case said that he is a happy and capable person [laughs], then I called them… those nurses said that oh my, we who have cared for Kauko, we know he is not capable nor always happy. I asked why is that written there? She said something like that it is just the typical thing [laughs] to say in a medical case summary*[…] *One morning they sent him for an ultrasound in [a central hospital], because they thought he was in good shape and capable. In the morning, I went to see him and they [the nurses] said to me, “No, no, they took him to [the central hospital] to have an ultrasound screening.” I said oh my god, if I would have known I’d have gone with him*[…]* Eventually he came back in the evening. The next morning they called me, “Can you come here?” They can’t make Kauko to go to X-ray because he is so angry. And I asked why he has to go to X-ray. And they said, well, his hand is hurt. I was terrified, the whole hand was blue all over and swollen, all the way to the fingertips. And he had these awful compression marks. I asked what terrible thing has happened to him, and they just go, “We don’t know.” That this has to have happened there at [central hospital].*

In the following extract, Sirpa, a person with memory disorder, describes her fear and insecurity*.* The actions by the professionals raise worry that her husband Sauli and the formal carers will just “put” her in an institution, that is, nursing home or a similar place.

*Sirpa: I received some brochures, and I started thinking that now Sauli is putting me to some institution, to some group home. I was so disappointed, so disappointed*[…]* And all the papers we receive, when reading those, I really start to feel that now they are just looking for the way how to put me there, there with the people with memory disorders upstairs, I don’t want to go there.*

*Sauli: Nobody’s going to take you there.*

*Sirpa: No no, but it’s like, there’s been indications of it. Isn’t so?*

*Sauli: No, I don’t understand.*

The data show that it has remained unclear to Sirpa what her husband and formal carers have agreed. Thus, Sirpa fears that she cannot influence her own care because others decide for her. This extract shows that the fear of being transferred in an institution is not just about influencing one’s *care* but moving to an institution would mean a total change of *life*. A move to an institution means living with strangers and the end of the life one is used to. Hence, not being able to influence one’s care can have a major and even irreversible impact on a person’s life.

## Discussion

We distinguished three themes describing different ways people with memory disorders and their carers influence formal care: Acquiescence, negotiating care, and taking control. The fourth theme describes the lack of influence. According to these descriptions, both people with memory disorders and their spouse carers have ways to influence formal care, but the latter had more ways to do so. Both spouse carers and people with memory disorders could submit to the decisions and actions of the professionals, but they did also take control of the situations and made their own decisions. In addition to these approaches, spouse carers negotiated with formal carers to influence care: an option which seemed not to be available for people with memory disorders. At times, the interviewees brought forward that they saw formal carers’ decisions and actions as inconsistent, or that the rationale for their actions was difficult to follow, but they had limited opportunities to have influence on these actions and decisions.

Of the four themes, *taking control* required strong agency and the most active and autonomous decision-making (Bosco et al., 2019; Smebye et al., 2012). Especially, the spouse carers took the power to decide whether to accept the care offered to the person being cared for (Lambotte et al., 2019). We recognize similar findings than Mikkola (2005) that spouses insisted on their right to define the terms and conditions of use of the services. It is noteworthy though that in our study, this also applied to some of those with memory disorders. Interestingly, these probably the strongest descriptions of active participation and autonomy were often related to conflict or disagreement related to mismatched expectations (Tuijt et al., 2020) or needs. While taking control requires strong agency, *acquiescence* was associated with a rather weak agency. In acquiescence, people are sort of pseudo-autonomous decision-makers as someone else has a strong impact on them (Bosco et al., 2019).

To influence care, different types of negotiations between formal carers and spouses were common, including shared decision-making (Bosco et al., 2019; Smebye et al., 2012), convincing, and persuasion. *Negotiating care* (also in Mikkola, 2005) as shared decision-making is in line with good care practices that emphasize treating people as individuals who have their own history, wishes, and values (Fazio et al., 2018). Yet, spouse carers often described situations where negotiations include persuasion, as negotiations did not proceed in mutually satisfactory ways, and the actual decision-making was largely in the hands of the formal caregiver. Acknowledging agency and the personal history of people with dementia, and including them in care planning, are the key elements in person-centered dementia care (Fazio et al., 2018; Kitwood & Bredin, 1992). This has been shown to reduce neuropsychiatric symptoms and depression and to improve the quality of life (Kim & Park, 2017). Nonetheless, people with memory disorders did not provide these accounts. We do not know whether they just did not express these situations in the interviews or if they had not experienced them. It is also possible that because the respondents with memory disorders had the help of the spouse available, they delegated the negotiations to the spouses (Smebye et al., 2012), or the formal and informal carer excluded—perhaps unintentionally—the person with memory disorders from negotiations.

As in Lambotte et al. (2019), our results showed how family carers can enact their agency for the behalf of the person with memory disorder. In our findings, they provided a larger share of the accounts concerning influencing care. However, when the spouse carer privately negotiated with the formal carer on matters about which the person with memory disorder disagreed, the spouse’s agentic power increased at the expense of the person with memory disorder. Agency always involves power relations. If the spouse overruns the power of the person with memory disorder to have a say in his/her care, then it is the spouse who diminishes their agentic power and not necessarily the professional carer. While the spouse carers’ actions aim to protect their loved ones from harming themselves or other people, this exclusion, or “noninvolvement,” positions people with cognitive impairment as incompetent (Smebye et al., 2012), and the symptoms of cognitive decline are used as justification for the exclusion (Tuijt et al., 2020).

To enact agency, capacity and personal resources are required (Marshall, 2005) at least to some extent. The view that enacting agency is about rational goal-oriented decision-making expressed with eloquent narration effectively denies agency of people with memory disorders, particularly if their conduct is not seen as rational by ordinary standards (Boyle, 2014; Kontos, 2005). However, agency is possible only within the confines of existing social and physical structures (Marshall, 2005). Agency of people with memory disorders is relational and dependent on the actions of the spousal carers and formal carers alike, as well as on social circumstances and the resources provided for the actors (see, Burkitt, 2016). Therefore, we should not attribute limited influence only to individual capacity as structural issues in the health and social care services play a crucial role. Our interpretation is that two structural issues weakened people’s possibilities to influence care: power imbalance and complex service system.

First, when dealing with formal carers, the encounters are characterized by power imbalance that has implications for the ability to make decisions and influence personal circumstances (Burkitt, 2016). Formal care providers have control over types of care that cannot be provided by lay individuals. They have the pressure to provide care at certain financial costs which limits the availability of services. Power imbalance restricts the client’s participation in care, which might lead to distrust and to encounters where crucial information is not shared with professionals (Berry et al., 2017). Power imbalance does not need to cause these adverse outcomes as by acknowledging the personhood of the person with dementia (Fazio et al., 2018; Kitwood & Bredin, 1992) and by person-centered communication (Downs & Collins, 2015), the individual needs and preferences can be taken into account in formal care decisions. A lack of communication and appropriate information often seems to underlie when people feel unable to influence care. Hence, adequate communication is needed for involving them to formal care and making the rationale behind the decisions clearer (Tuijt et al., 2020).

Second, there is an urgent need to clarify the roles and responsibilities of different actors in care and provide timely information of different forms of support. In connection to this, frequent contacts with at least one trusted formal carer would secure continuity of care and assist people to navigate the service system. In Peel and Harding (2014), informal carers found the care service system as “huge maze” where one has to fight for the services one needs. Navigating the system was time-consuming, unpredictable, and often more difficult than the caring they undertook. Our results include similar descriptions: Situations where one had to seek care persistently and situations where people were unsure whether they were entitled to care. Addressing these malfunctions in the service system is of paramount importance as the number of home-dwelling people with memory disorders has increased due to reduced access to institutional care and an increase in aging-in-place care policy (OSF, 2017).

## Strengths and limitations

Our data included descriptions provided by people with memory disorders and their spouse carers; this can be seen as a limitation as well as a strength. The common understanding of agency has been criticized for being language-centered (Boyle, 2014) and too heavily based on cognitive and intellectual abilities (Kontos, 2004). Even though our study showed that cognitive decline makes a person’s own involvement more difficult (also in Bosco et al., 2019; Lambotte et al., 2019), our study demonstrated that the abilities of people with memory disorders are very diverse and strongly connected to each individual’s personal situation. Our data show that people with memory disorders can express their preferences, even if the way they express themselves might be less determined or fluent than of people with intact cognition. It is unclear to what degree they had lower possibilities to influence over their own care or if they were less keen or less able to describe such situations during the interviews. Also, the presence of a spouse carer may limit their expression because they assume the spouse will do it “better.” This study is based on these one-off interviews—a second round of interviews could have produced more in-depth discussion and clarification regarding the influence on care. In addition, we acknowledge that the possibilities to influence care vary according to the service type. However, we wanted to approach this topic specifically from the perspective of the dyad. Hence, we did not differentiate between the encounters in different services as different professionals and sectors mix and intertwine in people’s descriptions as well as sometimes in practice.

The participants were involved with third-sector organizations, which meant they were likely relatively active. There are many less active people and those without family carers. These people are likely in the most vulnerable positions, but they are also the hardest to reach for research purposes. Our findings cannot, therefore, be generalized to all people with memory disorders or their spouse carers in Finland. Still, as this study gave voice to people with memory disorders and concentrated on influence over care in a broader sense than limiting it to decision-making, it offers a novel view to ways home-dwelling people with memory disorders and their family carers influence the care they receive from health and social care providers. In addition, our findings underscore the importance of discussing care with people with memory disorders before the condition is severe (Whitlatch et al., 2005) or at the severe stage, taking advantage of alternative ways to interpret their wishes than by verbal communication alone (Boyle, 2014; Kontos, 2004).

## Conclusions

This study identifies both the limitations and diversity in the ways how people can influence the care they receive from formal providers. It also demonstrates the importance of family carers in the care of people with memory disorders. People with memory disorders and their family carers are often in a disadvantaged position as they lack power over the health and social care decision-making during the illness, which is often guided by structural factors rather than the patient’s preferences or person-centered care. To reach the situation of shared decision-making and taking into account the patient’s preferences, the care of home-dwelling people with memory disorders requires clarification of the service structure, clearer and more equal communication between the different agents, and a change in attitudes that takes greater account of the views of people with memory disorders.
